# Learning more about hepatitis E virus

**DOI:** 10.7554/eLife.87047

**Published:** 2023-03-22

**Authors:** Altaira D Dearborn, Ashish Kumar, Joseph Marcotrigiano

**Affiliations:** 1 Structural Virology Section, Laboratory of Infectious Diseases, National Institute of Allergy and Infectious Diseases, National Institutes of Health Bethesda United States

**Keywords:** Hepatitis E virus, viral hepatitis, virus infection, replication, Viruses

## Abstract

A domain in the ORF1 polyprotein of the hepatitis E virus that was previously thought to be a protease is actually a zinc-binding domain.

**Related research article** LeDesma R, Heller B, Biswas A, Maya S, Gili S, Higgins J, Ploss A. 2023. Structural features stabilized by divalent cation coordination within hepatitis E virus ORF1 are critical for viral replication. *eLife* **12**:e80529. doi: 10.7554/eLife.80529.

Hepatitis E virus (HEV) is a single-stranded, positive-sense RNA virus that is spread by fecal-oral transmission. Although infection is usually self-limiting, it can result in death via acute liver failure. The World Health Organization estimates that HEV causes 20 million infections and 44,000 deaths per year, particularly among expectant mothers (WHO, 2022). The genome of the HEV contains three open reading frames that produce: (i) an enzyme that helps the virus to replicate itself; (ii) a capsid protein for the protein shell that surrounds the newly replicated viruses; (iii) a viroporin that helps the new viruses to escape from cells that have already been infected so that they can infect other cells.

In HEV, translation of the first open reading frame (ORF1) produces a polyprotein that contains seven domains. Multi-domain polyproteins are also made by other viruses, including HIV, hepatitis C virus, Chikungunya, Dengue, SARS coronavirus, rubella, influenza, and polio. In most other viral families, this polyprotein is then cleaved into individual proteins by enzymes called proteases that derive from the virus or its host (Yost and Marcotrigiano, 2013). Although the domain organization of the HEV ORF1 polyprotein is similar to other viruses (Figure 1), it is not clear if ORF1 undergoes cleavage. Previous studies have suggested that ORF1 contains a domain that acts as a protease, with a cysteine residue (Cys483) and a histidine residue (His590) acting as the catalytic sites. However, while Cys483 is highly conserved, His590 is not, and there is little evidence that this domain (which is called a putative papain-like cysteine protease, or pPCP for short) operates as a protease.

**Figure 1.**
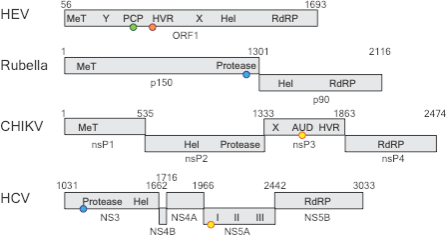
Comparing four RNA viruses. The seven domains of the ORF1 polyprotein for the hepatitis E virus (HEV; top) are shown schematically and compared to polyproteins from rubella, Chikungunya (CHIKV), and hepatitis C virus (HCV). All four viruses contain a helicase enzyme (Hel) and an RNA polymerase enzyme (RdRp). Rubella, CHIKV and HCV contain proteases, but LeDesma et al. have shown that the PCP domain in HEV that was previously thought to be a protease is a zinc-binding domain. The locations of the zinc-binding motifs are represented by coloured spheres: green for 6Cys (HEV); orange for HisGluHis (HEV); blue for 3Cys1His (Rubella and HCV), yellow for 4Cys (CHIKV and HCV). MeT: methyltransferase; Y: Y-domain; PCP: papain-like cysteine protease; HVR: hypervariable region; X: macro-domain; AUD: alphavirus unique domain; NS/nsP: non-structural protein.

Now, in eLife, Alexander Ploss and colleagues at Princeton University – including Robert LeDesma as first author – report the results of experiments which shed light on the role of the pPCP domain (LeDesma et al., 2023). Their results indicate that this domain – while necessary for replication of the virus – is not a protease, but rather a structural organization and localization domain. Moreover, they also show that Cys483 facilitates zinc binding rather than being a catalytic site for a protease.

If the pPCP domain were a protease, LeDesma et al. hypothesized that it would be possible to rescue protease-defective mutants by expressing pPCP in trans, so they generated cell lines that expressed either the wild-type ORF1 polyprotein, two mutant ORF1 polyproteins (called C483A and Pol(–)), or the wild-type pPCP domain alone. The next step was to transfect each of these cell lines with a reporter RNA in which ORF1 was either wild type or one of the mutants. Their results suggest that the pPCP domain is either not a protease or not proteolytically active in isolation.

The researchers then turned their attention to the residue Cys483. If this residue were part of a protease catalytic site then it, and no other cysteines in the pPCP domain, would support replicase activity. However, alanine and triple-alanine mutation indicated that six of the eight cysteines in the PCP domain are critical for replicase activity.

Since there is no protease, they investigated what the pPCP domain and the residue Cys483 might do. LeDesma et al. noticed that a six-cysteine motif within the domain was similar to other proteins that may bind bivalent metal cations. Using inductively coupled plasma mass spectrometry and confocal microscopy, the researchers observed that the mutation C483A reduced the ability of the domain to bind zinc ions, and also resulted in ORF1 being unable to localize in the nucleus.

Like all the best science, this work raises more questions than it answers. Zinc-binding domains with unique folds have been identified in a number of positive-sense RNA viruses (Shin et al., 2012; Tellinghuisen et al., 2004; Tellinghuisen et al., 2005; see coloured circles in Figure 1), and if the six cysteines of the pPCP domain bind zinc, the structure will be novel. A transcriptional activator in yeast called Gal4 is the foundational example of a six-cysteine, zinc-binding motif (Hong et al., 2008), but the six-cysteine pattern of the pPCP domain does not align well with the sequence or structure of Gal4, which again suggests a novel structure.

In Chikungunya, a viral protease digests the polyprotein to generate a functional replication complex (Tan et al., 2022). In the absence of a protease, how is this achieved in HEV? Zinc-binding domains often function as dimers or as repeat domains. Does pPCP structurally organize the other domains within a single copy of the ORF1 polyprotein, or does it organize multiple ORF1s? Many zinc-binding domains bind double-stranded nucleic acids, and the six-cysteine region in pPCP has several basic residues that could facilitate this.

Given the significant effect that HEV infection has on human health, more information about ORF1 domain organization and function can assist in the development of drugs to combat disease.
